# Functional Cakes: The Effect of Acorn Flour and Hydrocolloids and Emulsifier on Texture, Nutritional and Sensory Properties

**DOI:** 10.1002/fsn3.70703

**Published:** 2025-07-29

**Authors:** Mohsen Ebrahimi Hemmati Kaykha, Ali Forouhar, Hamed Saberian

**Affiliations:** ^1^ Department of Food Science and Technology, College of Agriculture Isfahan University of Technology Isfahan Iran; ^2^ Department of Agro‐Industrial Waste Processing Academic Center for Education, Culture and Research (ACECR) at IUT Isfahan Iran

**Keywords:** acorn flour, cupcake, functional properties, hydrocolloids

## Abstract

Cakes are widely appreciated for their desirable taste and soft texture, making them an excellent vehicle for incorporating health‐promoting nutrients such as polyphenolic compounds into the diet. This study explores the use of acorn flour (AF), a natural source of bioactive compounds, as a partial replacement (30% w/w) for wheat flour (WF) in cake formulations aimed at improving nutritional value. To enhance functional properties, hydrocolloids and the emulsifier DATEM were included at five concentrations (0%, 0.1%, 0.3%, 0.5%, and 0.9% w/w based on AF content). Physical and chemical analyses were performed to assess the impact of these ingredients on product quality. The highest porosity, indicative of superior aeration and a softer crumb, was observed in the formulation containing DATEM. In contrast, the greatest hardness occurred in the AF‐only sample without any additives. Incorporating hydrocolloids and emulsifier into 30% AF cakes enhanced important textural attributes, including hardness, cohesiveness, springiness, and chewiness. The addition of AF also increased redness and total phenolic content, contributing to a potentially higher antioxidant capacity. Sensory evaluation showed that the formulation with 30% AF and xanthan gum (XG) achieved the highest overall acceptance, highlighting its favorable balance of texture and taste. These findings support the application of AF as a promising functional ingredient in the bakery industry, offering potential for the development of novel health‐oriented products that align with consumer demand for nutritious, clean‐label alternatives.

## Graphical abstract

This study suggests that the incorporation of acorn flour at a concentration of 30% into cake batter may improve the nutritional and health benefits. Furthermore, CMC demonstrated a superior balance of the assessed technological properties, including hardness, springiness, volume, and porosity, in comparison to the other gums tested.

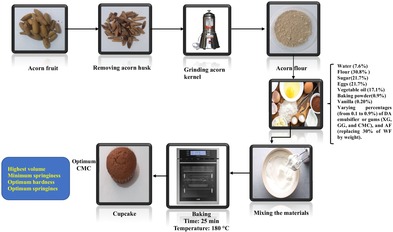

## 1 Introduction

The products obtained from cereals are among the first foods known to human societies and play a great role in the economy and nutrition of the people of the world, especially in developing countries. Recent studies have demonstrated AF's versatility in gluten‐free bakery applications, improving antioxidant activity, mineral content, and structural properties in breads and muffins (Beltrão Martins et al. 2020; Levent and Aktaş 2024; Szabłowska and Tańska 2025). The main components of the cake are wheat flour (WF), oil, sugar, and eggs, and the selection and substitution of any of these ingredients can change the structure, flavor, taste, and quality of the product (Mamat et al. 2025).

In recent years, the popularity of bioactive foods has led to an increase in innovation in food formulations, leading to improved consumer health (Goranova et al. 2019). On the other hand, adding new edible components to bakery products has created a pleasant aroma, color, and taste, significantly affecting consumers' acceptance (Złotek 2018). Many studies have been done over several years about replacing part of the flour in the cake formulation with compounds from other sources of plants (Zareh et al. 2016), flax flour (Ayoubi 2018), lentil flour (Khalil Zadeh et al. 2016), sorghum flour (Naghipour et al. 2013), and the mixture of corn flour and rice flour (Yanpi et al. 2018). Some researchers and managers focused on enhancing food products' quality and nutritional value. Incorporating low‐cost but nutritionally valuable ingredients can be an effective and economical strategy to improve the nutritional profile of the food products while maintaining or reducing production costs. However, achieving this goal requires maintaining the quality of the food products at an optimal level and ensuring it remains attractive to consumers. One of the low‐cost food ingredients is acorn flour (AF).

The oak tree belongs to the family Fagaceae and the genus *Quercus*. Seven genera and more than 900 oak species have been found in temperate and subtropical regions. The tree grows mainly in Europe, North Africa, North America, the Middle East, and Asia, and has two types: deciduous and evergreen (Ofcarcik and Burns 1971). Acorn (the fruit of oak trees) has been considered the main food of people in these regions until about the 20th century (Sasani et al. 2023). AF has gained attention for its potential to boost the nutritional value of baked goods, including bread and pastries (Torabı et al. 2020). It offers several health benefits, such as being gluten‐free, maintaining a high level of dietary fiber and minerals, and providing natural antioxidants like tocopherols and polyphenols, which support overall wellness (Akcan et al. 2017; Al‐Rousana et al. 2013; Beltrão Martins et al. 2020). Despite these advantages, integrating AF into bakery and confectionery products is not without its difficulties. Its baking qualities tend to fall short when compared to standard flours, resulting in noticeable changes in the physical qualities of the finished products. For instance, baked bread with oak flour frequently displays greater density, smaller volume, reduced porosity, and a firmer consistency (Hrusková et al. 2019; Purabdolah et al. 2020; Skendi et al. 2018). Moreover, AF contains various flavonoids, including quercetin, gallic acid, catechin, chlorogenic acid, and pyrocatechol (Taib et al. 2020). Remarkably, the antioxidant activity of the acorns was preserved even after heat treatment was used to reduce tannin content (Rakić et al. 2007). A key characteristic of AF is its ability to absorb large amounts of water, which can be beneficial for dough handling and can lead to improved product texture. Conversely, this high water retention ability may hinder dough processing, reducing its elasticity and stretchability. Consequently, products containing AF often have a compact structure, decreased volume, and are harder overall (Szabłowska and Tańska 2025).

Compounds in acorn fruits vary among different species, so the amount of these compounds, which include carbohydrates, fiber, protein, and fat, has been determined to be in the ranges of 41.52%–78.83%, 13.11%–51.76%, 2.08%–4.94%, and 1.78%–3.21%, respectively (Skendi et al. 2022). Few studies have been conducted on the replacement of WF with AF. Parseh et al. (2023) conducted a study to optimize a new sponge cake recipe by replacing WF and sugar with AF (0%–30%) and acorn syrup (AS) (0%–100%). They found that replacing WF with AF increased dough density, decreased baking percentage, and reduced cohesiveness, lightness (L*), and redness (a*) (Parseh et al. 2023). Korus et al. (2015) also reported that replacing AF with potato starch at an amount of 20% had a positive effect on the volume and physical and textural properties of gluten‐free bread, as well as slowing down the staling process. However, as the replacement amount increased (more than 20%), the quality characteristics of the bread decreased (Korus et al. 2015). Unfortunately, the application of acorns in the human diet is not widespread, although it has a good potential as an alternative functional food (Sardão et al. 2021). A cake batter is an oil‐in‐water emulsion in which fat particles are irregularly located in the aqueous phase (Abbaszadeh et al. 2023). Hydrocolloids, which are often known as gums, are hydrophilic polymers of plant, microbial, animal, or synthetic origin and generally contain hydroxyl groups, and in some cases polyelectrolytes, and are usually naturally present or added to foods. The most important properties of hydrocolloids include creating viscosity (consistency or gel) and binding of water, but some other properties include the stability of emulsions, the prevention of ice recrystallization, and organoleptic properties (Kohajdová, Karovičova, and Schmidt 2009).

The use of hydrocolloids, such as guar gum (GG), carboxymethyl cellulose (CMC) and xanthan gum (XG) or some emulsifiers such as Datem (DA) improves the quantitative and qualitative characteristics of the final product (Zomorrodi and Faramarzi 2020). Despite its nutritional benefits and functional potential, the application of AF in cake formulations remains relatively underexplored. Most existing research has concentrated on breads and gluten‐free products, where AF has demonstrated advantages in enhancing nutritional profiles but has also posed challenges regarding texture and volume (Beltrão Martins et al. 2020; Szabłowska and Tańska 2021). In contrast, limited attention has been given to its role in cake systems, which rely on fat–air emulsions and structural integrity. Notably, there is a lack of comprehensive data on how hydrocolloids and emulsifiers may mitigate the functional drawbacks of AF—particularly its lack of gluten and negative impact on batter aeration. Addressing this gap, the present study investigates the combined effects of three hydrocolloids (CMC, GG, and XG) and the emulsifier DATEM on the quality characteristics of cakes containing 30% AF. This work aims to generate practical insights for formulating functional bakery products by optimizing the synergy between alternative flours and structural enhancers.

## 2 Materials and Methods

Local producers from Isfahan, Iran, kindly provided the raw materials. They were then stored at room temperature in polyethylene bags until the following experiments.

### 2.1 Batter Preparation

Critical components in the formulation of the cake included the following: 7.6% water, 30.8% flour, 21.7% sugar, 21.7% eggs, 17.1% vegetable oil, 0.9% baking powder, 0.20% vanilla, and varying percentages (from 0.1% to 0.9%) of DA emulsifier or hydrocolloid, such as XG, GG, CMC, and AF (replacing 30% of WF by weight). Sugar, eggs, and vanilla were combined by kitchen mixer (Moulinex, France) for 5 min before adding the oil. Baking powder and flour were incorporated after 2 min of mixing, and the mixture was continued until it formed a uniform batter. After 25 min at 180°C in an electric oven, 65 g of batter was poured into each 8.5 cm diameter baking mold. Two replicate baking experiments were conducted. The samples were carefully preserved in polyethylene containers for all analyses.

### 2.2 Cake Volume

The specific volume of the cake samples was measured according to the protocol outlined in AACC Method 10‐05.01, using the rapeseed displacement method. First, a box with a known volume was filled with rapeseeds to determine their bulk density. After the cake samples had cooled to room temperature, a weighed sample was carefully placed into a volumetric container pre‐filled with rapeseeds. The seeds were gently leveled to eliminate air gaps and ensure uniform packing around the sample. The mass of the container with the seeds and cake, along with the volume of displaced rapeseeds, was recorded. The volume of displaced seeds calculated using the bulk density was considered equal to the volume of the cake (Majzoobi et al. 2013). Finally, the specific volume (cm^3^/g) was calculated by dividing the cake's volume by its weight using Equation (1):

(1) $$\text{Specif}\;\mathrm{ic}\;\text{volume}\;\left({\mathrm{cm}}^{3}/g\right)=\frac{\text{Volume}}{\text{Weight}}$$

### 2.3 Firmness and Texture Profile Analysis

The texture of cakes made with AF was analyzed using a texture analyzer (Santam, STM1, Iran) equipped with a 36‐mm‐diameter cylindrical probe and employing a texture profile analysis test (TPA). The assessment was conducted at a test speed of 1.0 mm/s, exerting 50% compression (double‐cycle) on samples (2.5 × 2.5 × 2.5 cm) from the central region, with a trigger force of 0.05 N. Several textural characteristics were assessed, including hardness, springiness, cohesion, gumminess, and chewiness. Hardness: Defined as the maximum force recorded during the first compression cycle, indicating the resistance of the cake to initial deformation. Cohesiveness: Calculated as the ratio of the area under the force–time curve of the second compression to that of the first compression. This parameter reflects the internal bonding strength and the ability of the sample to withstand repeated deformation. Springiness: Determined the distance that the sample recovers its height during the second compression. It expresses the elasticity or the tendency of the sample to return to its original shape after deformation. Calculated as the product of hardness and cohesiveness (Gumminess = Hardness × Cohesiveness). Chewiness: Computed as the product of hardness, cohesiveness, and springiness (Chewiness = Gumminess × springiness). This composite parameter represents the energy required to masticate the sample to a consistency suitable for swallowing (Tasnim et al. 2020).

### 2.4 Color Measurements

An image processing approach was used to analyze the sample color properties (L*, a*, b*, ∆*E* and Hue). Cake samples were placed in the box intended for image capture. A digital camera (Canon PowerShot A540) was positioned 30 cm above the samples, and fluorescent lights inside the box were activated 5 min before capturing the image. The cakes' crust and crumb images were collected and processed using Image J software version 1.40. The L* value represents the brightness of the samples, ranging from 0 for dark colors to 100 for the lightest hue. Positive a* values represent red, whereas negative values suggest green. Positive and negative b* values indicated yellow and blue colors, respectively (Forouhar et al. 2023).

∆*E* also indicates the color difference of the samples with the control sample, which was calculated from Equation (2). In this formula, ∆*L* was the difference in brightness, ∆*b* was the difference in blue‐yellow, and ∆*a* was the difference in red‐green of each sample with the control sample:

(2) $$∆E=\sqrt{{\left(∆L\right)}^{2}+{\left(∆a\right)}^{2}+{\left(∆b\right)}^{2}}$$

In addition, the hue angle (Hue) indicates the type of color, and as it approaches zero, the color tends towards red, and as it approaches 90, the color goes towards yellow, and the numbers 180 and 270 indicate green and blue, respectively. Therefore, the hue angle was calculated based on Equation (3):

(3) $$\mathrm{Hue}=\tan^{-1}\left(\frac{b*}{a*}\right)$$

### 2.5 Porosity

The internal structure of the samples (sample porosity) was analyzed using Image J software version 1.44 from the National Institutes of Health in Bethesda, MD, USA. The samples were cut with a sharp knife to generate a cross‐section, which was then scanned and saved in a 2048 × 1536 DPI resolution using an HP2000 flatbed scanner. The brightness/contrast option in Image J was used to improve image clarity and enhance the quality of the cropped image. The photos were then processed using an intermodal filter to reduce image noise. Porosity characteristics were examined using binary images. Initially, the images were converted into 8‐bit grayscale format. Subsequently, binary conversion was performed to distinguish between solid and void regions. In the binary images, bright and dark pixels represent different structural features of the cake, with dark pixels generally corresponding to air voids. The ratio of dark to total pixels was then analyzed as an estimate of the cake porosity. This ratio, which reflects the relative amount of open structure in the cake matrix, was ultimately reported as a percentage to facilitate comparison across samples. As expected, a higher proportion of dark pixels indicates a more porous texture (Amani et al. 2022).

### 2.6 Determination of Total Phenolic Content (TPC)

The TPC was determined using the method of Pasqualone et al. (2019) and a spectrophotometer (UV/VIS). 0.2 g of sample with 4 mL of acidic methanol (1 mL of hydrochloric acid, 80 mL of methanol, and 10 mL of water) was mixed at room temperature for 2 h. The 200 μL of the extract was added to 5.2 mL of Folin–Ciocalteu reagent, and then sodium carbonate and distilled water were added to it. The tubes containing the resulting mixture were completely covered and left at room temperature for 30 min. Finally, the absorbance of the samples was measured at a wavelength of 760 nm by a spectrophotometer (Pasqualone et al. 2019). The TPC was calculated using a gallic acid calibration curve, and its results were reported as gallic acid equivalents (GAE) g/g of dry plant matter.

### 2.7 Optimization

While other textural properties—such as cohesiveness, chewiness, and gumminess—are indeed relevant for comprehensive texture analysis, hardness and springiness were chosen for this study due to their direct and measurable impact on consumer perception, as validated by sensory panels. The optimized cake formulation can improve product quality and consumer satisfaction. Sequential quadratic programming was employed to adjust the gums and DA content to optimize cake formulation. This process was performed using the ‘fmincon’ function in MATLAB's software, with the objective function designed to minimize hardness while maximizing both the springiness and volume of the samples. Finally, we calculated a score for each gum type by normalizing the hardness, springiness, and volume values and applying a weighted sum method with equal weights. The gum with the highest score was identified as the optimal choice for achieving the desired balance of properties in the cake formulation.

### 2.8 Sensory Evaluation

Twelve semi‐trained panelists evaluated the sensory attributes (taste, color, texture, and general acceptability) using a 5‐point hedonic scale. The panelists were given samples labeled with random codes and asked for their opinions on the taste, color, texture, and overall acceptability of samples on a scale from 1 to 5. The scale varied from 1, denoting strong dislike, to 5, indicating strong liking (Amerine et al. 2013).

### 2.9 Statistical Analysis

An ANOVA was performed to evaluate significant differences in the results (*p* < 0.05) utilizing SPSS software. Tukey multiple comparison tests were used to examine the variations in the sample results (SPSS Inc., NJ, USA).

## 3 Results and Discussion

### 3.1 Characteristics of AF

The results of the evaluation of the AF compounds are shown in Table 1. Chemical analysis conducted on the AF obtained from Quercus species has confirmed that acorns are a high‐energy food. They are rich in carbohydrates and fats, but have a relatively low protein content. However, environmental and genetic factors can significantly affect the fat and protein content of acorns (Vinha et al. 2016). The results of this research were consistent with those of (Pasqualone et al. 2019). Martins et al. (2022) reported the composition of AF in their study as follows: moisture content of 8.15 ± 0.03, ash content of 1.61 ± 0.01, total protein content of 4.28 ± 0.27, total fat content of 11.39 ± 0.53, and carbohydrate content of 0.57 ± 0.53 per 100 g (Martins et al. 2022). A higher concentration of fiber and minerals (ash) was observed in gluten‐free biscuits supplemented with acorns than in the control samples (Korus et al. 2017; Torabı et al. 2020).

**TABLE 1:** Chemical composition of acorn flour.

Compound	(g/100 g)
Ash content	1.3 ± 0.1
Total protein	3.7 ± 0.3
Carbohydrates	80.5 ± 0.2
Fiber	0.6 ± 0.05
Moisture	7.2 ± 0.03
Fat	6.7 ± 0.1

### 3.2 Cake Volume

**FIGURE 1:**
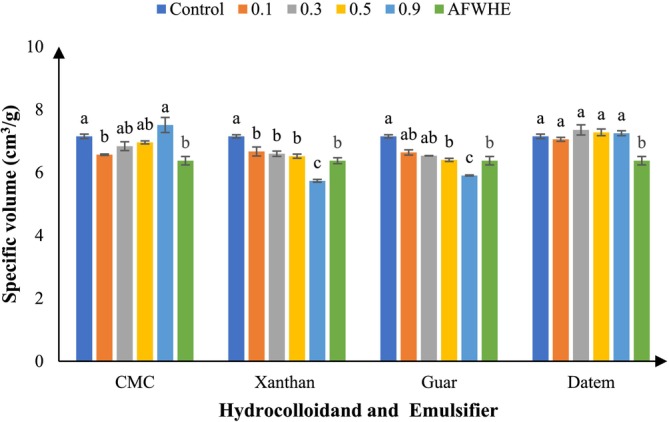
Specific volume characteristics of samples containing different levels of hydrocolloid and DATEM. Statistical differences (*p* < 0.05) among treatments are indicated by different letters above the bars.

The cake volume reflects the gases, mainly carbon dioxide and ammonia, generated by baking powder during baking, along with structural changes. Moisture absorption and additives in the formulation also influence this property (Baeva, Panchev, and Terzieva 2000). Figure 1 shows the effect of replacing 30% AF with WF and adding different hydrocolloids and DA emulsifier on the volume of samples. The cake volume decreased in the samples containing 30% AF compared to the control sample (no AF). The cake volume increased by increasing the CMC concentration from 0.1% to 0.5% compared to the AFWHE sample in the cake formulation. This is due to the hydrocolloid network provided by the CMC, which gives strength to the gas cells of the dough. Expansion occurs during baking, which ultimately reduces gas loss through the cake. This improves its texture. Therefore, the volume of the cake increases significantly as the CMC increases.

In addition, a similar decrease in volume was observed in samples containing 0.9% XG and GG. When XG was added to samples containing 30% AF, it was observed that there was a significant difference between the samples containing hydrocolloid and the control sample (*p* > 0.05). Also, when different concentrations of this hydrocolloid were added to cakes containing 30% AF, a significant difference was observed with the control sample (*p* < 0.05). Based on statistical analysis, the difference between the samples containing DA emulsifier and the control sample was not significant (*p* > 0.05), although these samples differed significantly from the sample with AFWHE. Therefore, the DA emulsifier helped to mitigate the reduction in cake volume caused by AF. Some hydrocolloids or emulsifiers can help to distribute the air cells evenly in the batter, thus increasing the volume of the cake. Also, hydrocolloid and emulsifiers can increase the water holding capacity (WHC), thereby influencing the batter body and better entraining air (Salehi 2020). Seyhun, Sumnu, and Sahin (2003) noted that emulsifiers promote small gas cell formation in batter, aligning with this study, where DA emulsifier significantly increased cake volume (Seyhun, Sumnu, and Sahin 2003). Szabłowska and Tańska (2025), in a study, stated that with increasing the proportion of AF, the volume of released gases increased (up to 140% in the sample containing 30% AF), while the volume of remaining gases decreased (up to 92% in the sample with 50% AF), which was consistent with the results of this study (Szabłowska and Tańska 2025).

### 3.3 Texture Profile Analysis

#### 3.3.1 Hardness

**FIGURE 2:**
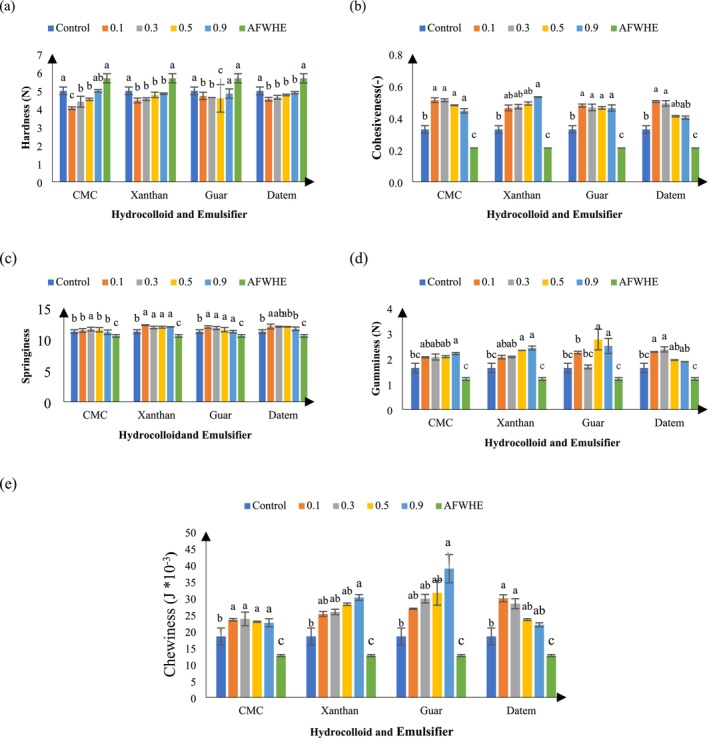
Textural properties of samples containing different levels of hydrocolloid and DATEM. Statistical differences (*p* < 0.05) among treatments are indicated by different letters above the bars.

Food texture, as one of the most important quality characteristics of the product, plays an important role in overall consumer acceptance. Figure 2 illustrates the effect of replacing 30% AF with WF and adding emulsifiers and hydrocolloid on the texture of cupcake samples. Textural properties were evaluated based on the hardness, cohesiveness, springiness, gumminess, and chewiness of the cupcakes. As shown in Figure 2a, replacing 30% AF with WF increased the hardness of all samples, while adding hydrocolloid and emulsifiers decreased the hardness level of the samples. The addition of different concentrations of hydrocolloids (CMC, XG and GG) and DA emulsifier reduced the hardness of AF‐enriched samples. Also, samples containing 0.9% concentration of CMC and GG were not significantly different from the control sample (*p* > 0.05). Water‐soluble hydrocolloids with high molecular weights can form gels and thicken aqueous systems. The softening effect of different hydrocolloids is attributed to their ability to retain moisture in the cake formulation and prevent it from migrating to the starch, where it could lead to crystallization. In addition, the inclusion of hydrocolloid increases the viscosity of the batter (Hojjatoleslami and Azizi 2015), which may help to prevent air loss from the batter. Furthermore, Noorlaila et al. reported that high concentrations of hydrocolloid, in combination with other thickening agents, resulted in an excessive increase in batter viscosity. This significant increase in viscosity was strongly associated with increased cake hardness (Noorlaila et al. 2017). In this study, the hardness of samples containing 0.9% hydrocolloid was consistent with these findings.

Khoshdouni Farahani (2021) investigated the physicochemical, textural, and sensory properties of cocoa sponge cake formulated with XG. This research showed that the hardness of the samples was reduced by adding XG at concentrations of 0.2 and 0.3 (Khoshdouni Farahani 2021). CMC forms a three‐dimensional network that can bind water molecules within the system. It forms and provides a barrier layer during heating, reducing water loss and oil absorption (Andrew 2004). Sciarini et al. (2012) reported that the addition of CMC hydrocolloid to the dough of gluten‐free bread is effective in reducing the hardness of the bread core, increasing the amount of water absorption, maintaining the moisture of the bread tissue, delaying staleness, and extending its shelf life (Sciarini et al. 2012).

#### 3.3.2 Cohesiveness

Cohesiveness indicates the internal resistance of the food structure and its level depends on the intramolecular interactions of the formulation components. The effect of adding 30% AF, hydrocolloids (CMC, GG, and XG), and DA emulsifier on the cohesiveness of cake samples is illustrated in Figure 2b. In Figure 2b it is clear that the addition of various concentrations of CMC to cakes containing 30% AF resulted in a significant increase in cohesiveness. However, as the concentration of CMC increased, the cohesiveness decreased with a significant difference from the control sample (*p* < 0.05). The results of adding different concentrations of XG to cakes containing 30% AF showed that the cohesiveness increased with the addition of this hydrocolloid, with a significant difference from the control sample only at a concentration of 0.9% (*p* < 0.05). Gums also increase the viscosity of the batter. They strengthen the walls of the gas cells of the product and increase the resistance of the cake to the pressure exerted by the probe of the texture measuring instrument at high hydrocolloid consumption levels (Arozarena et al. 2001). In this regard, Turabi and colleagues (2008) reported that an XG‐GG blend without emulsifier increased the stiffness of the cake texture due to the thickening of the walls around the air bubbles in the core of the cake (Turabi, Sumnu, and Sahin 2008). Gómez et al. (2008) found that as the amount of chickpea flour in the formulation increased, other textural indices, such as adhesiveness, springiness, and elasticity decreased, and hardness, gumminess, and cohesiveness increased (Gómez et al. 2008). Consistent with the current findings, De La Hera, Oliete, and Gómez (2013) similarly showed that the cohesiveness, hardness, and springiness of cake samples decreased as the amount of oat flour in the formulation increased (De La Hera, Oliete, and Gómez 2013).

#### 3.3.3 Springiness

**FIGURE 3:**
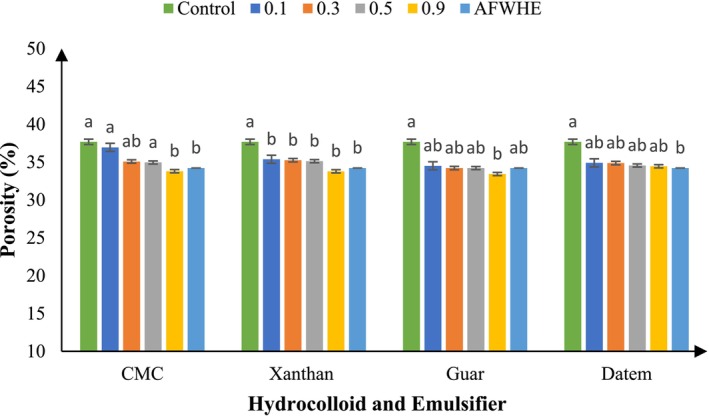
Porosity of samples with varying hydrocolloid and DATEM levels. Statistical differences (*p* < 0.05) among treatments are indicated by different letters above the bars.

Springiness is a critical quality attribute in cakes, representing their capacity to recover their initial form after the removal of a deforming force (Salehi et al. 2016). This parameter serves as a key indicator of crumb elasticity and is closely associated with consumer perceptions of freshness and structural integrity. The addition of CMC at 0.3% significantly enhanced springiness in cakes containing 30% AF. CMC is well‐documented for its water retention properties and its role in stabilizing air cells, thereby promoting a more aerated and flexible crumb structure (Shanina et al. 2019). However, increasing the CMC concentration to 0.9% resulted in diminished springiness. This reduction may be attributed to excessive batter thickening, which restricts gas cell expansion during baking, ultimately yielding a denser and less elastic texture. Elevated batter viscosity has been shown to hinder gas cell development, limiting the formation of uniform air pockets and resulting in reduced crumb porosity and elasticity (Narsimhan 2012). As demonstrated in Figure 3, this effect is evident in the decreased porosity of the cake samples.

Similarly, XG contributed to a statistically significant improvement in springiness compared to the control (*p* < 0.05). However, no significant differences were observed among varying XG concentrations (0.1%, 0.3%, and 0.5%), suggesting a saturation effect at lower incorporation levels. This phenomenon may arise from XG's inherently high viscosity, which can dominate the system's rheology even at minimal concentrations, thereby limiting further structural enhancements with additional increments (N'gouamba et al. 2021).

In contrast, the incorporation of DATEM, at 0.1%, 0.3%, and 0.5%, led to a consistent and significant increase in springiness (*p* < 0.05). DATEM is widely utilized in baking formulations due to its ability to strengthen the gluten network and improve gas retention during leavening and baking. It interacts synergistically with both gluten and starch components, enhancing dough extensibility and improving crumb elasticity (Xiujin et al. 2007). In gluten‐deficient systems, such as those supplemented with acorn flour, DATEM compensates for structural deficiencies by reinforcing elasticity. The observed decline in springiness with increasing AF content aligns with prior research findings. Acorn flour, being naturally gluten‐free and rich in dietary fiber, increases batter viscosity due to its high water absorption capacity. In addition, the inability of the proteins in this flour to create a network and retain gas was identified as a reason for reducing springiness. Increasing the percentage of AF in the sample formulation resulted in these effects (De La Hera, Oliete, and Gómez 2013). As regards the importance of gluten in springiness, it has been shown that an increase in substitution results in a decrease in springiness. The high amylase content of acorn starch affects the springiness due to its tendency to retrograde.

#### 3.3.4 Gumminess and Chewiness

In TPA, gumminess refers to the energy required to decompose a semi‐solid food for ingestion, while chewiness refers to the energy required for oral digestion of a solid food (Szczesniak 1991). The results related to gumminess are given in Figure 2d. Different changes were observed after adding different hydrocolloids and emulsifier to a cake formulation. Adding hydrocolloid (CMC and XG) to the cake ingredients increased the gumminess. No significant difference was observed between samples with different concentrations of CMC and XG (*p* > 0.05).

In cake samples containing GG, gumminess increased after adding 0.1% concentration, but its value decreased in the sample containing 0.3% concentration. When different concentrations of DA emulsifier were added to cake samples containing 30% AF, the gumminess value initially increased. Subsequently, it decreased by adding a higher amount of DA in the sample formulation. The chewiness results are shown in Figure 2e. According to the chewiness results, this parameter increased when CMC was added up to 0.5%. However, no significant difference was observed between samples with different concentrations of CMC (*p* > 0.05). The addition of XG at different concentrations increased this parameter. In fact, the sample with a concentration of 0.9% was significantly different from the control samples (*p* < 0.05). In addition, adding DA emulsifier to cakes led to an increase in chewiness. Figure 2d,e show that gumminess and chewiness were drastically decreased by substituting 30% AF without using hydrocolloids and emulsifier in the cake samples. Hydrocolloids encourage the gelatinized starch granules to stick together.

These sticky interactions are believed to reinforce their structure. Increasing their volume by swelling and absorbing water can increase the stresses exerted on them and promote the solubilization and exudation of amylose. Using AF as a substitute for base flour in cakes with a spongy structure, leavened with yeast or baking powder, leads to variations in characteristics similar to those observed in bread (Molavi et al. 2015). On the other hand, sponge‐fat cakes exhibit a smaller volume, higher density, and a decrease in the texture‐related features of the product. Furthermore, an increase in hardness, cohesiveness, gumminess, adhesiveness, springiness, and chewiness is also observed (Molavi et al. 2015).

### 3.4 Color Measurements

From a technological standpoint, the color of samples is critical because it has a significant impact on product appeal and customer satisfaction. Food color is the first quality parameter evaluated by the consumer and influences the final product acceptance. Therefore, when substances are added to food, there should be no adverse change in color. Currently, the most common models for measuring food color are Lab or L*, a*, and b* (Pathare et al. 2013). Table 2 shows the results in terms of the amount of color change in the center and surface of the cake samples.

**TABLE 2:** Changes in L*, a*, b*, ∆*E*, and hue of cake samples containing different concentrations of hydrocolloids and emulsifier.

Parameter	Sample	Center				Surface			
		CMC	XG	GG	DA	CMC	XG	GG	DA
L*	Control	41.92 ± 1.36^c^	41.92 ± 1.36^c^	41.92 ± 1.36^c^	41.92 ± 1.36^c^	27.35 ± 0.68^a^	27.35 ± 0.68^a^	27.35 ± 0.68^a^	27.35 ± 0.68^a^
	0.1	47.97 ± 1.36^b^	51.42 ± 2.05^a^	51.20 ± 2.05^a^	43.79 ± 1.09^b^	50.53 ± 1.36^c^	52.40 ± 1.36^c^	41.13 ± 1.36^b^	42.72 ± 0.51^b^
	0.3	48.31 ± 3.42^b^	51.16 ± 0.68^a^	49.54 ± 0.68^a^	45.35 ± 2.18^b^	58.31 ± 1.36^cd^	57.41 ± 0.00^c^	53.25 ± 0.00^c^	44.20 ± 1.81^b^
	0.5	49.07 ± 2.87^a^	52.56 ± 2.05^a^	49.37 ± 0.68^a^	46.36 ± 1.64^a^	60.45 ± 1.36^d^	58.84 ± 0.68^cd^	56.00 ± 0.68^c^	53.39 ± 0.55^c^
	0.9	51.70 ± 1.03^a^	52.28 ± 2.05^a^	49.44 ± 0.0^a^	49.03 ± 0.54^a^	65.08 ± 0.42^d^	61.86 ± 2.73^d^	61.28 ± 2.73^d^	60.43 ± 0.39^c^
	AFWHE	44.57 ± 1.36^b^	44.57 ± 1.36^b^	44.57 ± 1.36^b^	44.57 ± 1.36^b^	39.15 ± 0.68^b^	39.15 ± 0.68^b^	39.15 ± 0.68^b^	39.15 ± 0.68^b^
a*	Control	−6.93 ± 1.09^a^	−6.93 ± 1.09^a^	−6.93 ± 1.09^a^	−6.93 ± 1.09^a^	5.41 ± 1.09^a^	5.41 ± 1.09^a^	5.41 ± 0.72^a^	5.41 ± 1.09^a^
	0.1	6.85 ± 1.09^b^	6.44 ± 1.09^b^	6.85 ± 0.0^b^	7.03 ± 1.09^b^	12.02 ± 1.03^c^	11.96 ± 0.51^bc^	13.82 ± 1.43^d^	12.80 ± 1.60^c^
	0.3	6.76 ± 0.10^b^	6.96 ± 1.09^b^	7.16 ± 0.65^b^	7.04 ± 1.09^b^	10.01 ± 0.00^b^	11.19 ± 0.00^b^	12.60 ± 1.43^c^	13.22 ± 1.90^d^
	0.5	7.44 ± 0.38^b^	6.83 ± 0.00^b^	6.93 ± 0.00^b^	6.83 ± 3.28^b^	9.94 ± 0.00^b^	10.31 ± 0.00^b^	12.96 ± 1.43^c^	13.25 ± 1.54^d^
	0.9	6.44 ± 1.09^b^	6.87 ± 0.00^b^	7.22 ± 1.64^b^	6.38 ± 0.00^b^	8.05 ± 0.17^b^	10.21 ± 0.00^b^	9.95 ± 0.47^b^	10.60 ± 0.71^b^
	AFWHE	5.86 ± 1.09^b^	5.86 ± 1.09^b^	5.86 ± 1.09^b^	5.86 ± 1.09^b^	10.08 ± 0.51^b^	10.08 ± 0.51^b^	10.08 ± 0.51^b^	10.08 ± 0.51^b^
b*	Control	25.15 ± 0.56^a^	25.15 ± 0.56^a^	25.15 ± 0.56^a^	25.15 ± 0.56^a^	27.42 ± 0.57^a^	27.42 ± 0.57^a^	27.42 ± 0.57^a^	27.42 ± 0.57^a^
	0.1	16.97 ± 0.56^b^	19.16 ± 0.56^b^	18.87 ± 1.13^b^	19.26 ± 1.13^b^	25.79 ± 0.56^ab^	24.10 ± 1.13^ab^	24.31 ± 1.13^c^	22.50 ± 0.57^c^
	0.3	17.50 ± 0.56^b^	18.28 ± 0.56^b^	18.44 ± 1.13^b^	18.84 ± 1.70^b^	24.69 ± 1.70^ab^	25.01 ± 0.56^ab^	25.61 ± 1.70^a^	24.04 ± 0.56^b^
	0.5	17.48 ± 1.70^b^	18.46 ± 0.00^b^	18.49 ± 2.83^b^	18.74 ± 1.70^b^	23.93 ± 1.13^b^	27.53 ± 0.00^ab^	26.12 ± 0.00^ab^	27.1 ± 0.57^a^
	0.9	18.13 ± 1.13^b^	18.44 ± 0.56^b^	17.33 ± 0.56^b^	18.63 ± 0.56^b^	22.83 ± 0.57^c^	28.53 ± 0.00^a^	27.41 ± 1.13^ab^	26.78 ± 0.56^a^
	AFWHE	18.77 ± 0.56^b^	18.77 ± 0.56^b^	18.77 ± 0.56^b^	18.77 ± 0.56^b^	22.87 ± 0.12^c^	22.87 ± 0.12^c^	22.87 ± 0.12^c^	22.87 ± 0.12^c^
∆*E*	Control	00.00 ± 0.00^f^	00.00 ± 0.00^f^	00.00 ± 0.00^f^	00.00 ± 0.00^f^	00.00 ± 0.00^f^	00.00 ± 0.00^f^	00.00 ± 0.00^f^	00.00 ± 0.00^f^
	0.1	262.42 ± 2.16^b^	224.24 ± 1.36^d^	238.78 ± 1.13^d^	231.44 ± 1.13^c^	69.52 ± 1.70^a^	78.97 ± 0.57^a^	94.18 ± 0.57^a^	94.18 ± 57^a^
	0.3	252.26 ± 0.36^c^	249.12 ± 1.09^a^	251.15 ± 0.56^b^	238.40 ± 1.12^a^	59.57 ± 0.36^d^	69.27 ± 1.13^b^	80.87 ± 1.13^c^	89.27 ± 0.57^b^
	0.5	272.39 ± 0.59^a^	244.34 ± 1.64^c^	243.86 ± 1.36^c^	234.87 ± 1.70^b^	65.80 ± 0.56^b^	55.51 ± 0.56^d^	87.34 ± 1.70^b^	87.60 ± 0.56^c^
	0.9	237.49 ± 1.13^d^	245.57 ± 0.56^b^	268.92 ± 0.56^a^	226.25 ± 0.56^d^	65.76 ± 0.12^c^	58.78 ± 0.56^c^	54.54 ± 0.12^d^	60.42 ± 0.56^d^
	AFWHE	206.89 ± 0.65^e^	206.89 ± 0.65^e^	206.89 ± 0.65^e^	206.89 ± 0.65^e^	54.31 ± 0.56^e^	54.31 ± 0.56^e^	54.31 ± 0.56^e^	54.31 ± 0.56^e^
Hue	Control	105.28 ± 0.17^a^	105.28 ± 0.17^a^	105.28 ± 0.17^a^	105.28 ± 0.17^a^	78.73 ± 1.13^a^	78.73 ± 1.13^a^	78.73 ± 1.13^a^	78.73 ± 0.57^a^
	0.1	68.38 ± 1.36^d^	71.52 ± 0.56^c^	70.08 ± 1.13^c^	69.84 ± 0.56^d^	65.44 ± 0.56^3^	63.57 ± 0.57^f^	60.36 ± 0.56^f^	60.36 ± 1.09^f^
	0.3	68.57 ± 0.56^d^	69.18 ± 0.56^f^	68.76 ± 1.09^e^	69.41 ± 0.56^e^	67.76 ± 0.12^c^	65.86 ± 1.13^e^	63.76 ± 0.12^d^	61.22 ± 1.54^e^
	0.5	66.77 ± 1.70^e^	69.65 ± 0.00^d^	69.38 ± 0.56^d^	69.80 ± 1.03^d^	67.40 ± 0.56^c^	69.43 ± 0.00^c^	63.54 ± 0.00^e^	63.74 ± 0.00^d^
	0.9	70.64 ± 0.56^c^	69.56 ± 0.56^e^	67.31 ± 0.65^f^	71.16 ± 1.43^c^	70.54 ± 0.56^b^	70.24 ± 0.56^b^	70.14 ± 0.57^b^	68.27 ± 1.36^b^
	AFWHE	72.76 ± 0.12^b^	72.72 ± 0.12^b^	72.72 ± 0.12^b^	72.72 ± 0.12^b^	66.51 ± 1.09^d^	66.51 ± 1.09^d^	66.51 ± 1.09^c^	66.51 ± 1.09^c^

*Note:* Values are expressed as mean ± standard deviations, *n* = 3; different letters (a, b, c, d, e and f) in each row show significant difference at *p* ≤ 0.05.

The color characteristics of the center of the samples are shown in Table 2. In terms of the color results at the center of the sample, specifically the redness and yellowness index, it is evident that adding different hydrocolloids and DA emulsifier did not affect these color characteristics of the samples. Instead, AF is the dominant factor affecting the color. This explanation is substantiated by the observation that samples containing AF exhibited no significant differences. However, there is a significant color difference when compared to the control sample.

The color characteristics of the sample surface are shown in Table 2. When different concentrations of hydrocolloids (CMC, XG, and GG) were added to cake samples, the L* of the cake surface increased significantly compared to the control sample (*p* < 0.05). Additionally, adding various concentrations of DA emulsifier increased the amount of a* significantly, which differed from the control sample (*p* < 0.05). When different hydrocolloids and DA emulsifier were added to cake samples containing 30% AF, the b* value of all samples decreased.

Furthermore, by adding varying concentrations of this hydrocolloid into cakes containing 30% AF and assessing the colorimetric parameters L* and a* at the center of the cakes, a significant difference was noted between the samples containing CMC and the control sample (*p* < 0.05). However, no significant difference was observed in the b* parameter at concentrations of 0.1 and 0.3 when compared to the control sample (*p* > 0.05).

In cake samples containing 30% AF, the addition of varying concentrations of XG led to a significant variation in all three parameters (L*, a*, and b*) at the center of the samples when compared to the control sample (*p* < 0.05). However, in the surface factor, only the parameters L* and b* exhibited a significant difference, while parameter a* for the surface factor did not show any notable differences between the control sample and the various concentrations of XG (*p* > 0.05).

According to the data presented in Table 2, adding GG into cakes containing 30% AF resulted in statistically significant variations when compared to the control sample. These differences were evident in three parameters (L*, a*, and b*) at the surface and in two parameters (L* and a*) at the center across all concentrations. However, concerning the surface parameter b*, a significant difference from the control sample was observed only at a concentration of 0.1, with no significant variations noted at other concentrations (*p* > 0.05).

Adding different concentrations of DA to cake samples containing 30% AF resulted in significant differences in all parameters (L*, a*, and b*) in the center of the samples compared to the control sample. This emulsifier also affected the surface parameters L* and a*. However, for parameter a* related to the surface factor, the addition of emulsifier at concentrations of 0.5 and 0.9 did not show any significant difference when compared to the control sample (*p* < 0.05).

The results also showed that adding different concentrations of (CMC, XG and GG) as well as emulsifier DA to cake samples containing 30% AF resulted in a significant difference in the parameter (∆*E*) in the center and surface of the samples.

The results also indicated that adding various concentrations of (CMC, XG, and GG) and emulsifier DA to cake samples containing 30% AF decreased the Hue angle at both the surface and center of the cake.

Sharadanant and Khan (2003) reported that adding locust bean gum to bread dough formulation resulted in a lighter and more desirable bread crust color. At the same time, carrageenan led to darker crusts with undesirable characteristics (Sharadanant and Khan 2003). The increased lightness of the bread crust can be attributed to the addition of hydrocolloids, which influence water distribution and subsequently affect both the caramelization process and the Maillard reaction. Previous studies by Mandala et al. (2008) have shown similar findings, where incorporating hydrocolloids resulted in a lighter bread crust (Mandala et al. 2008). An overlay can be explained by the fact that the gums do not strongly alter the cake's color intensity but can influence the distribution of colorants within the batter and stabilize colorants rather than directly impacting color. So, as can be seen, the redness of samples containing the 30% AF has a higher a*. DA emulsifier primarily affects texture and batter structure rather than color. The slight change in redness with the addition of DA emulsifier is likely due to the improvement of the dispersion of colorants and other ingredients (from AF) in the cake batter, and the improved emulsification may result in a more uniform distribution of colorants that have been protected from thermal degradation and change.

It is evident that adding AF resulted in darker cakes because AF is naturally darker than the other ingredients in the mix. The measurement of parameter a* (redness‐greenness) in the sample surface showed an increase in the redness of the cakes with the addition of AF. Additionally, the measurement of the b* value (yellow–blue) indicated a decrease in the yellowness of the cakes when AF was added. Similar findings were observed in Barbari bread, where the use of AF resulted in a decrease in brightness and yellowness and an increase in redness (Majzoobi et al. 2013).

### 3.5 Porosity

The number and uniform distribution of gas cells in product directly affect the degree of porosity in the product's texture (Alencar et al. 2015). The results related to porosity are given in Figure 3. Adding different concentrations of CMC (0.1%–0.5%) improved the porosity of the AF‐enriched samples. The concentration of 0.9% CMC was significantly different from the control sample (*p* < 0.05), but not significantly different from the sample AFWHE (*p* > 0.05). Thus, the concentration of CMC lower than 0.9% is appropriate for the improvement of this property. After combining different concentrations of GG with cake samples containing 30% AF, no significant differences were observed between the control samples, with the exception of the 0.9% concentration (*p* > 0.05). Also, adding the XG did not change the porosity of the sample containing the AF. The results also showed that adding different concentrations of DA did not show any significant difference with the control sample. However, replacing AFWHE caused a significant difference in the porosity with the control sample (*p* < 0.05).

**FIGURE 4:**
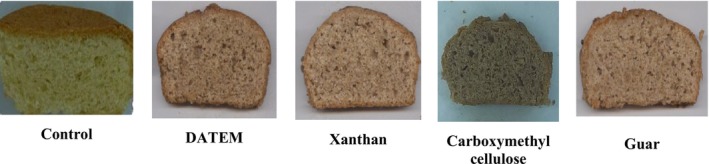
Porosity of cakes containing 30% AF with a concentration of 0.3% of different hydrocolloids and emulsifier.

Figure 4 shows the porosity of cakes containing 30% AF with a concentration of 0.3% of different hydrocolloids. The cake's porosity is influenced by the gas cells' stability in the dough and the prevention of their fusion, along with ensuring uniform gas bubble size in the final product (Rathnayake et al. 2018). If the batter has a low viscosity, it cannot hold the trapped bubbles. Consequently, these bubbles will quickly rise to the surface and be eliminated by the heat during baking. As a result, the porosity of the cake will be reduced (Mokhtari et al. 2019). The porosity of the product decreases with the addition of AF, resulting in less uniform distribution due to a lower gluten content in the formulation, as indicated by the obtained results (Avazsufiyan et al. 2014).

Sahraiyan et al. (2014) found that the addition of *Balung shirazi* gum at a concentration of 1% in sorghum gluten‐free bread led to a decrease in porosity. This reduction was attributed to the disruptions in the fermentation process and a decrease in gas cell formation (Sahraiyan et al. 2014). By using an emulsifier and diminishing the interfacial tension of the liquid component within the dough or batter, uniform dispersion of small air bubbles will occur throughout the entire dough. Consequently, during the cooking process, the air will be effectively released from these bubbles, creating a cake with a porous texture (Sakiyan et al. 2004).

### 3.6 Interrelationships Among Cake Quality Parameters

The incorporation of hydrocolloid gums in cake formulations significantly influences structural and textural properties as shown in Table 3. This analysis examines Pearson correlation coefficients to elucidate the relationships between porosity, specific volume, hardness, and springiness in samples with added XG, GG, CMC, and DA. As seen in Table 3, porosity‐volume synergy occurs with minimal textural impact. In samples with XG, a strong positive correlation between porosity and volume (*r* = 0.822, *p* = 0.012), indicating that increased air entrapment directly enhances cake expansion. This aligns with its known ability to stabilize gas cells via viscous network formation (Mills et al. 2003). Notably, hardness showed no significant correlation with porosity (*r* = −0.003) as Balak et al. previously reported (Balak et al. 2015), or volume (*r* = −0.109), suggesting XG's textural neutrality is likely due to its shear‐thinning behavior, which balances firmness and aeration. A moderate negative trend between springiness and porosity (*r* = −0.563, *p* = 0.147) implies that higher porosity may slightly reduce elasticity (Asamoah et al. 2023), although it is notsignificant. In the samples with GG, a positive porosity‐volume relationship (*r* = 0.734, *p* = 0.038), consistent with its high water‐binding capacity promoting gas retention (Asamoah et al. 2023) However, it uniquely displayed a strong negative correlation between volume and hardness (*r* = −0.897, *p* = 0.002), possibly due to disrupted starch‐gluten matrices. Additionally, harder cakes with GG were significantly less elastic (*r* = −0.731, *p* = 0.039), suggesting that this fibrous network may limit recoverable deformation (Hojjatoleslami and Azizi 2015). According to Table 3, like GG, CMC showed a strong inverse volume‐hardness relationship (*r* = −0.853, *p* = 0.007), reinforcing that CMC stabilizes air cells and improves sample volume while reducing structural density (Ammar et al. 2020). Negative volume‐hardness correlations (GG, CMC) likely arise from diluted structural networks, whereas DA's emulsifying action reinforces matrix strength. The porosity‐volume trend was positive but non‐significant (*r* = 0.602, *p* = 0.115), potentially due to sample variability. Springiness remained unaffected, indicating CMC's limited role in elastic recovery. In contrast to other gums, DA produced a significant positive correlation between volume and hardness (*r* = 0.768, *p* = 0.026). This suggests DA strengthens protein‐lipid interfaces, enabling simultaneous expansion and firmness (Hojjatoleslami and Azizi 2015). Porosity and hardness also trended positively (*r* = 0.631, *p* = 0.093), hinting at a unique microstructure combining air‐cell stability with dense crumb. DA excels in denser textures.

**TABLE 3:** Pearson's correlation analysis results for different deferent parameters of sample quality for the overall acceptance.

			Correlations			
			Porosity	Volume	Hardness	Springiness
Sample with xanthan gum	Porosity	Pearson correlation	1	0.822^a^	−0.003	−0.563
		Sig.		0.012	0.994	0.147
	Volume	Pearson correlation		1	−0.109	−0.49
		Sig.			0.797	0.217
	Hardness	Pearson correlation			1	−0.503
		Sig.				0.204
	Springiness	Pearson correlation				1
Sample with Guar gum	Porosity	Pearson correlation	1	0.734^a^	−0.454	0.159
		Sig.		0.038	0.259	0.707
	Volume	Pearson correlation		1	−0.897^b^	0.685
		Sig.			0.002	0.061
	Hardness	Pearson correlation			1	−0.731^a^
		Sig.				0.039
	Springiness	Pearson correlation				1
Sample with CMC	Porosity	Pearson correlation	1	0.602	−0.599	−0.043
		Sig.		0.115	0.116	0.92
	Volume	Pearson correlation		1	−0.853^b^	0.279
		Sig.			0.007	0.504
	Hardness	Pearson correlation			1	−0.509
		Sig.				0.197
	Springiness	Pearson correlation				1
Sample with DATEM	Porosity	Pearson correlation	1	0.646	0.631	−0.580
		Sig.		0.083	0.093	0.132
	Volume	Pearson correlation		1	0.768^a^	−0.197
		Sig.			0.026	0.640
	Hardness	Pearson correlation			1	−0.295
		Sig.				0.477
	Springiness	Pearson correlation				1

^a^ Correlation is significant at the 0.05 level.

^b^ Correlation is significant at the 0.01 level.

### 3.7 Total Phenolic Content

**FIGURE 5:**
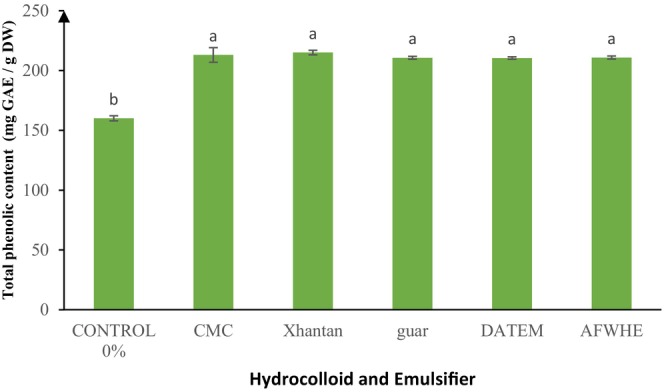
Total phenolic content (TPC) of samples containing varying concentrations of hydrocolloids and DATEM. Statistical differences (*p* < 0.05) among treatments are indicated by different letters above the bars.

Total Phenolic Content (TPC) is an important characteristic with the potential to promote consumer health. TPC of cake samples containing different hydrocolloids (CMC, XG, GG), DA emulsifiers, and 30% AF replacement is shown in Figure 5.

As shown in Figure 5, the addition of AF to the cake increased the total phenolic content in all samples, showing a significant difference compared to the control sample (*p* < 0.05). Additionally, there was a significant difference (*p* < 0.05) in phenolic compound levels between the 30% AF samples (both with and without hydrocolloid) and the control sample. The phenolic content in plant tissues is influenced by multiple factors, including genetics, sunlight, soil conditions, ripeness at harvest, and post‐harvest practices (Faller and Fialho 2009). According to the research conducted by Khameneh and Demirag (2021), the Iranian AF contains a phenolic composition of 323.16 (mg GAE/100) (Khameneh and Demirag 2021). Martins et al. (2022) found during their research that the amount of phenolic compounds in the AF was 16.79 (Martins et al. 2022). Several studies have shown that adding AF to biscuits significantly improved antioxidant activity and decreased peroxide value compared to control samples. The incorporation of AF into cake restricts oxidative alterations and enhances antioxidant activity. This is predominantly attributed to the abundant presence of antioxidant phenolic compounds in AF (Korus et al. 2017; Parsaei et al. 2018; Pasqualone et al. 2019; Torabı et al. 2020).

### 3.8 Optimization

The optimization of cake formulations involved the evaluation of different gums, including XG, GG, and CMC, as well as the emulsifier DA. Each additive's ideal concentration was determined by assessing its impact on the cakes' technological characteristics. The analysis revealed that the optimal concentration for XG was 0.23%, while GG exhibited its best performance at 0.21%. The most effective concentration for CMC was 0.26%, and DA demonstrated optimal results at 0.25%. These results emphasize the importance of precise concentration levels for each additive to achieve superior cake quality. The optimization of cake formulation was conducted based on the hardness, springiness, volume, and porosity values of samples to determine the optimal gum and DA percentages. Based on the results, CMC was identified as the best‐performing additive. These results reflect CMC's superior balance of the evaluated technological properties, including hardness, springiness, volume, and porosity, compared to the other gums tested.

### 3.9 Sensory Evaluation

After optimizing the cake formula, sensory evaluation was done on the samples. The effect of the type of treatments on sensory analysis, including (color, uniformity, softness, test, and overall acceptability) was analyzed for each treatment separately. As shown in Table 4, the control sample received the highest score among other samples. The cake made with AF had a brownish hue, attributed to the presence of reducing sugars in the flour. However, this did not negatively impact the evaluation of the panelists. In this parameter, the samples containing hydrocolloid (CMC, XG, GG) and DA emulsifier had a significant difference from the control sample (*p* < 0.05). The inclusion of AF reduced the cakes' flavor attributes. A significant difference was observed in this parameter between the samples containing CMC and GG and the control sample (*p* < 0.05). Still, no significant difference was observed between the control sample and the DA emulsifier (*p* > 0.05). The softness parameter showed that softness increases significantly with the addition of CMC, but it is not substantially different from the control sample (*p* < 0.05). The addition of XG and GG, as well as DA emulsifier to the cakes containing 30% AF and samples without hydrocolloid, significantly differed between the control sample and the sample containing CMC (*p* < 0.05). The uniformity and overall acceptability parameters showed that adding hydrocolloid (CMC, GG) and DA emulsifier to cakes containing 30% AF showed a significant difference between these samples and the control sample (*p* > 0.05). A literature review also revealed the effects of adding AF on the flavor of pastry products. It was reported that the addition of AF could have a negative impact on the taste and aroma of the final products (Korus et al. 2017; Menasra and Fahloul 2018; Molavi et al. 2015; Torabı et al. 2020), as well as their textural features, which are particularly important for cakes with a spongy structure (Molavi et al. 2015).

**TABLE 4:** Changes in color, uniformity, softness, test, and overall acceptability of cake samples containing different hydrocolloids (CMC, XG, and GG), DA emulsifier and replacing AFWHE.

Treatment	Color	Test	Softness	Uniformity	Overall acceptability
Control	4.90 ± 0.33^a^	4.70 ± 0.10^a^	4.50 ± 0.35^a^	4.90 ± 0.44^a^	4.90 ± 0.37^a^
CMC	3.90 ± 0.33^b^	3.90 ± 0.30^b^	5.60 ± 0.06^a^	3.80 ± 0.33^b^	3.80 ± 0.39^b^
XG	3.80 ± 0.20^b^	4.20 ± 0.10^a^	3.70 ± 0.31^b^	4.00 ± 0.23^ab^	4.00 ± 0.17^ab^
GG	3.90 ± 0.20^b^	3.70 ± 0.21^b^	3.80 ± 0.31^b^	3.30 ± 0.49^b^	3.30 ± 0.41^b^
DA	3.90 ± 0.22^b^	3.60 ± 0.33^b^	3.70 ± 0.25^b^	3.40 ± 0.40^b^	3.40 ± 0.23^b^
AFWHE	3.90 ± 0.33^b^	3.80 ± 0.10^b^	4.00 ± 0.31^b^	3.60 ± 0.44^b^	3.60 ± 0.37^b^

*Note:* Values are expressed as mean ± standard deviations, *n* = 3; different letters (a, b) in each row show significant difference at *p* ≤ 0.05.

## 4 Conclusion

Replacing 30% of wheat flour with acorn flour (AF) enhances the nutritional value of cakes, particularly by increasing total phenolic content. However, this substitution also presents challenges, such as increased hardness, reduced volume, lower porosity, and changes in sensory quality. The inclusion of hydrocolloids (CMC, XG, GG) and the DA emulsifier effectively counteracts these drawbacks, with CMC standing out as the most impactful due to its moisture‐retaining properties and ability to stabilize air cells and strengthen cake structure. The most significant textural improvements—including reduced hardness and enhanced springiness, chewiness, and gas cell distribution—were achieved at optimized concentrations: CMC at 0.26%, XG at 0.23%, GG at 0.21%, and DA at 0.25%. The inclusion of AF changed the cake's color and flavor; however, hydrocolloids and emulsifiers contributed to restoring its visual appeal and sensory acceptance. These findings suggest that incorporating optimized levels of CMC and DA emulsifier can improve the texture, springiness, and volume of cakes containing 30% AF, making the formulation more suitable for commercial‐scale production of fiber and polyphenol‐enriched cakes. Overall, these results highlight the potential of functional ingredients to create nutritionally enriched cakes without compromising quality or consumer satisfaction.

## Author Contributions

**Mohsen Ebrahimi Hemmati Kaykha:** data curation (equal), methodology (equal), resources (equal), software (equal), writing – original draft (equal). **Ali Forouhar:** data curation (equal), methodology (equal), validation (equal).

## Conflicts of Interest

The authors declare no conflicts of interest.

## Data Availability

The data that support the findings of this study are available on request from the corresponding author.
